# Family history intake: a challenge to personalized approaches in health promotion and disease prevention

**DOI:** 10.1186/s13584-015-0055-2

**Published:** 2015-11-20

**Authors:** Ronit Endevelt, Iris Goren, Tal Sela, Varda Shalev

**Affiliations:** University of Haifa, Welfare and Health Faculty, School of Public Health, Haifa, Israel; Maccabi Health Care Services, Tel Aviv, Israel; Nutrition Department, Health Ministry, Jerusalem, Israel

## Abstract

**Background:**

Family history is considered an essential, obligatory part of the primary physician’s intake interview. Including coded FH in a unified medical file can save expensive genetic tests and detect the early onset of diseases in young people who are not recommended to be screened routinely. The objectives of this study are to explore the frequency and point in time of recording the coded family history (FH) as a first step to increasing awareness of the importance of such information.

**Methods:**

All ICD-9 coded diagnoses of familial histories of disease (ICD-9 coded V16.0 – V19.8), including diseases related to gender, age, and indications of chronic diseases, were collected from the electronic medical records of patients ages 18 and above in Israel’s Maccabi Health Care system. The study was carried out in 2012 on the basis of coded data for 1.9 million Maccabi members, which were collected from 2004 through 2011.

**Results:**

Of the Maccabi members (the second biggest HMO in Israel covering 2 million people), only 10 % had FH coded documentation. FH was significantly more frequent for females than for males (13.5 % vise 10.1 %) and increased with age. About 10 % of the FH documentation occurred before any disease was diagnosed. The most frequent FH documentation was observed for cardiovascular disease, hypertension, and diabetes. In the case of cancer FH was more frequent in females, whereas in the case of males it was cardiovascular disease.

**Discussion:**

Family history is an easy tool and need to be coded and implimented in most visits in order to get the best information of the potential health and disease of the patients.

**Conclusions:**

FH frequency is very low and varies with gender and age. The literature suggests that implementing it routinely in primary care will improve health care. Further research is needed to identify the factors that impede primary care givers from complying with FH guidelines.

## Background

In the absence of affordable and accessible genomic exams (known as "Omics"), the family history intake (FH) should become a core element of clinical care, as it reflects shared genetic, behavioral, and environmental factors and is a means of eliciting stigmatizing information about suicide, mental illness, alcoholism, sexual orientation, and past trauma that patients might be reluctant to share [1]. Such histories provide insights into health related behavior and build a solid foundation of trust. However, while evidence points to the utility of family health history screening, providers still face difficulties in using this tool to make clinical decisions [1]. Several studies show that physicians often report that they collect family history information [2, 3] and value its contribution [4], but other studies using actual encounter data suggest that often family history is either not obtained or is under utilized in risk assessments [5–8]. Moreover, there is a paucity of literature about how family history information is captured in primary care.

Maccabi Healthcare Services (MHS) is a large Israeli health maintenance organization (HMO) providing primary care services through approximately 3,000 solo practices to 1.9 million beneficiaries throughout the country in 2012. MHS is a fully computerized organization. Information including patient records, billing systems, pharmaceutical dispensations, and chronic disease registries is housed in the organization’s central electronic warehouse DATA BASE These automated datasets have been previously described at length in previous studies [9–12]. During the physician/nurse patient encounter, all of the information collected is recorded electronically in either coded or free text fields. However, there are no specific guidelines for the collection and recording of the FH of the general public. Furthermore, in most cases if a FH is queried, it happens after the patient has been diagnosed with a chronic disease (7). Currently, Maccabi physicians do not receive any financial incentives to document FH, nor does Maccabi engage its physicians about their failure to do so.

Including FH in a unified medical file can save expensive genetic tests and detect the early onset of diseases in young people who are not recommended to be screened routinely and those whose gender or unique characteristics put them at risk for developing particular diseases. As a first step in increasing awareness of the importance and usefulness of FH information, the objective of this study was to explore the habits of Israeli physicians regarding the recording of coded family history. The search for FH was limited to coded electronic fields. Free text was not evaluated, as only coded FH is transferred to other medical staff. Our sample group was MHS’s primary care physicians whom we assessed with regard to their recording of the intervening variables of the patient’s age, gender, and diagnosis.

## Methods

We searched MHS' database for all information coded as family history (ICD −9-CM codes: V16.0 – V19.8) for 1.9 patients ages 18 and over between the years 2004 and 2011. We selected this time frame because in 2004 all of the physicians’ medical files were computerized. In addition, when such information was documented, we searched the chronic disease registries to investigate whether and when a diagnosis was noted. Diagnoses were categorized into several areas: cancer, cardiovascular disease (CVD), hypertension, diabetes, non-diabetes endocrine diseases, ophthalmic illness, psychiatric disorders, and miscellaneous areas. Demographic data included gender and age. Rates were calculated with the disease sub-category as the denominator. We looked for differences in gender because of the different in disease pattern through life.

## Results

Overall, only 12 % of MHS' members had coded FH documentation in their electronic records. In all of the disease sub-categories, FH documentation was significantly more frequent for females than for males (13.5 % vise 10.1 %). About 10 % of the FH documentation occurred before any disease was diagnosed. The most frequent FH documentation was observed for CVD, hypertension, and diabetes before the diagnosis of the diseases (Table 1).

**Table 1:** FHI rates by gender and main disease sub-categories [%]

Disease sub-category	Male	Female	P value	Total MHS
CVD	12,724 [19.8]	8,385 [20.5]	0.004	21,109 [20.1]
Cancer	3,652 [11.8]	6,441 [17.7]	<0.001	10,093 [15%]
Hypertension	23,496 [19.8]	28,789 [22.8]	<0.001	52,285 [21.4]
Diabetes	10,967 [21.1]	10,307 [23.1]	<0.001	21,274 [22.0]
No Disease	65,358 [8.3]	97,277 [11.8]	<0.001	162,635 [10.1]
All MHS	98,724 [10.1]	135,838 [13.5]	<0.001	234,562 [12.0]

**Fig. 1:**
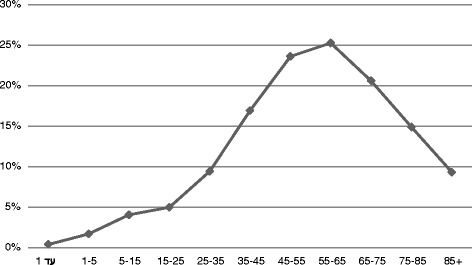
Percentage of the family history documentation by age group of the patients

**Fig. 2:**
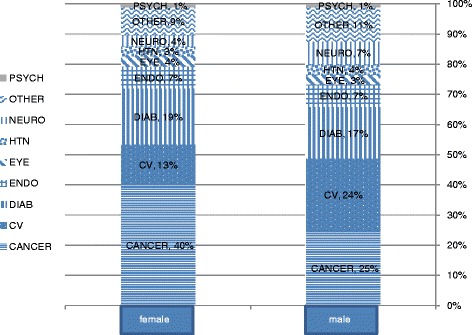
Prevalence of diagnosis of the family history in Maccabi HMS by gender

FH documentation increased with the patient's age, peaking at age 55–65 (Fig. 1). With regard to gender, we observed that more females (40 %) received FH documentation when cancer was diagnosed than did males (25 %). However, the opposite trend emerged for CVD (13 % for females and 24 % for males) (Fig. 2).

**Fig. 3:**
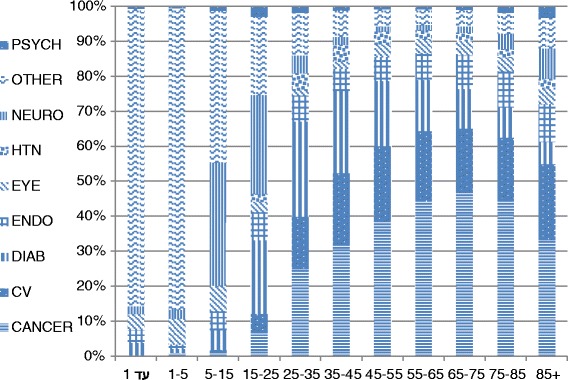
Family history of diagnoses by age groups. CV: Cardiovascular diseases; Diab: Diabetes; Endo: Endocrinology diseases; Eye: Eye diseases; HTN: Neuro: Neurological diseases; Psych: Psychiatric diseases

Figure 3 presents the distribution of all FH documentation by diagnoses and age group. FH documentation with a cancer diagnosis increased with age, whereas with a CVD diagnosis the frequency of FH documentation remained almost steady.

## Discussion

Our findings show the very infrequent documentation of coded FH information for both male and female members and for all disease sub-categories compared to other data related by the patient. Indeed, the documentation of FH with no diagnosis or prior to diagnosis occurred in only 10 % of the encounters between patients and physicians.

We found that more females (40 %) received FH documentation when cancer was diagnosed than did males (25 %). However, the opposite trend emerged for CVD (13 % for females and 24 % for males), maybe because of gender differences in the prevalence of the disease.

Given the current absence of affordable and accessible genetic tests and the immature state of clinical screening, a routine family history assessment may help identify those in primary care whose distinct familial disease pattern might indicate an underlying genetic predisposition that merits more extensive assessment [13]. Family health history is currently the most applicable genomic predictor for common, multifactor diseases, and can also highlight patterns that suggest a strong inherited susceptibility to a particular form of cancer or other disease. Both bloodline ancestry and shared environmental factors are important predictors of many diseases [14], and are important for increasing awareness about the risks of developing specific diseases, particularly for metabolic conditions, among those who underestimate their risk [15]. Primary care practitioners can also gain insight into patients' experiences of disease within the family, which are relevant to framing preventive advice [13].

However, despite the fact that many health professionals regard family history enquiry as a standard element of good medical care, and even when the disease is well known to be associated with family history, physicians often fail to collect such information [16, 17].

A study conducted in the US with Blue Cross and Blue Shield found that prompting family physicians about patients with family histories showing risks for six common conditions such as heart disease, stroke, diabetes, and breast, colorectal, or ovarian cancer did not seem to increase the identification or screening of these patients [18]. In contrast to our finding, a meta-analysis of the family history of cancer patients showed that physicians paid more attention to the FH of patients free of cancer than of those who were already suffering from the disease [19].

Family history should be taken every few years beginning with young people and updated frequently, because details can change over time. Possible strategies for promoting recording FH and updating it periodically include reminding the physician, other members of the clinical staff, and/or the patient to do so.

Time constraints are cited as one of the factors that limit the ability of physicians to comply with the recommendation to collect such data, often leaving them uninformed about the family’s history or lifestyle factors [20]. In addition, until recently, computerized systems with family history as an integral part of the patient’s medical history and decision making had not yet been standardized or widely available in a structured fashion, even though the U.S. Agency for Healthcare Research and Quality’s (AHRQ) expert panel concluded that systematic family history collection tools, some self-administered, are likely to improve the usual practices in primary care [21].

Another reason for the failure to collect FH is the ability of primary care physicians to interpret the information. According to the AHRQ panel, “In practical terms, the systematic collection of family history is linked with the interpretation of that information which in turn is linked to whether primary care providers take appropriate clinical action on the basis of the information collected” [21]. A qualitative study of 40 urban, suburban and rural physicians who had graduated from medical school between 1963 and 2000 noted that the doctors regarded a family history of cancer as important, but the process of obtaining this information and the content were not standardized. Major barriers to more focused use of this information included the limitations of the patients' knowledge about their family’s medical history, the time needed to clarify and interpret this information, and the lack of clear and accessible guidelines to assist in the collection, interpretation and management decisions for average, moderate and higher risk patients. In some populations, language and cultural barriers also made it more difficult to collect family histories [22].

The family history can be taken by any health professional if there is a united anamnesis medical file. Thus, once an up-to-date family history is recorded, ideally, decisions can be made that would help clinicians identify which aspects of the family history are clinically relevant. Using this data, health care professionals could determine the risk threshold that triggers earlier or more sensitive screenings for cancer and other chronic diseases or genetic evaluations for hereditary susceptibility to cancer and other diseases [23–27]. On the other hand, the collection of family history may also have the potential to increase the incidence of false-positive results and test-associated complications that are costly and potentially harmful [28, 29]. In times of limited resources, this possibility may affect the guidelines of health organizations and clinical practices.

In MHS, FH documentation is not a mandatory computer field, and the information is often written as free text rather than coded, so it is unidentified by computer algorithms. Moreover, given the absence of guidelines for FH, the system cannot raise “red flags” that would direct the physicians’ and other health staff’s attention to further inquiries into the family history.

Our results show that if FH was collected, it usually happened when the patients were older and already had a disease. However, FH is arguably more important for children and young adults because it indicates risk factors for illnesses such as cardiovascular disease and diabetes, allowing preventive measures to be taken as early as possible. In health care organizations such as MHS in which the family is a beneficiary unit, an electronic system linking a parent's morbidity to his/her children could be a step towards an efficient FH. Currently, in an effort to preserve the patient’s privacy, MHS follows a policy whereby physicians cannot access the records of their patients’ relatives. In addition, the family history should be discussed more consistently with new patients as well as with well-established patients [5]. To optimize the use of clinical time and resources, it is important to know when to update the family history of common diseases [29]. At this point we suggest that family nurses, dietitians and other health staff and the patients themselves by the personalize health record should play an important role in collecting FH and updating it once a year.

There are significant differences between female and male patients in the process of care, and physicians may be making medical decisions based on gender-related considerations [16]. There is evidence that female patients have longer visits, ask more questions, get more information, receive more counseling, send and receive more emotionally concerned statements, and appear more involved in the interaction than male patients [30]. In our case, FH was more frequent among female patients than male patients. Strategies for implementing knowledge about these gender differences are crucial for the delivery of gender-sensitive care and FH collection.

Our study has a number of limitations. The search for FH was limited to coded electronic fields. Free text was not evaluated. Only the coded data are accessible by specialists, hospitals etc. and this is critical in the new era of health information exchange. There may well be lots of family history intake in the free text, and this could be important for the primary care physicians' own use, and would be a useful focus for another study. However, it is not relevant to the current objectives which relate to what FH information will be available to all caregivers in the era.

Nevertheless, given the absence of an algorithm to assess free text, we consider such information less useful, unless the physician considered this information as valuable for the purposes for diagnosis. Furthermore, given that our goal was to explore the frequency of the presence of FH as a first step in increasing awareness of the importance of such information, we did not investigate the factors associated with the low rates of collecting family histories that are not coded. Such an investigation should be the subject of further research. Another important issue is the cultural obstacles from reaching out FH and there is a need to collaborate with other professions such as social workers in order to collect as much FH data available.

### Significance

The idea and findings in this paper are facing a big challenge to make a necessary step that physicians will obtain the family history of their patients. It can be applied for teaching, service and research purposes in healthcare management and education. It can also serve as a benchmarking tool for education and practice. The FH inquiry developed and initially validated in this study will contribute to fostering patient-centered medicine by utilizing electronic medical records. Including FH in a unified medical file can save expensive genetic tests and point to the early onset of diseases such as breast cancer in young people who are not recommended to be screened routinely. Raising awareness about the importance of routine inquiry into the patients and the current paucity of coded FH documentation’ family history may help improve their quality of care.

## Conclusions

The frequency of coded FH documentation is quite low and varies according to age and gender. Further research is needed to identify the factors that impede primary care givers from complying with family history guidelines and to assess the effectiveness various strategies for promoting more frequent FH documentation as was found in this study.

## Ethics approval

The study was confirmed by the institutional ethics committee of Maccabi Health Services.
